# Quarterly Table of Mortality in Great Britain

**Published:** 1844-11

**Authors:** 


*Quarterly table of Mortality in Great Britain.*—The deaths registered in the last quarter (ending June 30) amounted to 30.925, which is less by 7116 than the deaths of the previous quarter, and 1283 less than the average of the corresponding spring quarter in the five years 1838–42. Allowing for the increase of the town population, the mortality was ten per cent. below the average of the season.

The reduction in the mortality has been unequally distributed over the kingdom; but it has been most remarkable in the large manufacturing districts. Small-pox and scarlatina have been epidemic in the metropolis and elsewhere. The deaths in the quarter from small-pox were 425. The epidemics most frequently mentioned in the country districts are scarlatina, measles, small-pox, hooping cough, and typhus.

*Ibid* from *Ibid.*

